# Improving *in vivo* assays in snake venom and antivenom research: A community discussion

**DOI:** 10.12688/f1000research.148223.1

**Published:** 2024-03-15

**Authors:** Amy E Marriott, Nicholas R Casewell, Elliot Lilley, José-María Gutiérrez, Stuart Ainsworth

**Affiliations:** 1 Department of Infection Biology and Microbiomes, Institute of Infection, Veterinary and Ecological Sciences, University of Liverpool, Liverpool, England, L3 5RF, UK; 2 Centre for Snakebite Research and Interventions, Department of Tropical Disease Biology, Liverpool School of Tropical Medicine, Liverpool, L3 5QA, UK; 3 National Centre for the Replacement, Reduction and Refinement of Animals in Research, London, NW1 2BE, UK; 4 Instituto Clodomiro Picado, Facultad de Microbiología, Universidad de Costa Rica, San José, 11501-2060, Costa Rica

**Keywords:** Preclinical assays, Lethality, 3Rs, NAMs, Antivenom, Venom

## Abstract

On the 26 ^th^ January 2023, a free to attend, ‘improving *in vivo* snake venom research: a community discussion’ meeting was held virtually. This webinar brought together researchers from around the world to discuss current neutralisation of venom lethality mouse assays that are used globally to assess the efficacy of therapies for snakebite envenoming. The assay’s strengths and weaknesses were highlighted, and we discussed what improvements could be made to refine and reduce animal testing, whilst supporting preclinical antivenom and drug discovery for snakebite envenoming. This report summarises the issues highlighted, the discussions held, with additional commentary on key perspectives provided by the authors.

## Introduction

Snakebite envenoming is a Neglected Tropical Disease (NTD) that causes extensive mortality and morbidity worldwide. ^1^^,^^2^ Antivenom is the therapy of choice for treating envenomings and, if available, it can be a lifesaving treatment. However, antivenom is an unusual therapy as it is not routinely required to undergo the same level of scrutiny to demonstrate clinical effectiveness (i.e., clinical trials), as is routine with nearly all other medicines. ^3^ Instead, prediction of antivenom efficacy in humans, except for a very small minority of products, is inferred through murine preclinical testing. Since the landmark achievement of snakebite envenoming being reinstated as an NTD by the World Health Organization (WHO) in 2017, much attention has been rightly focused on improving antivenom access, availability, quality, and applicability. However, the actual methodology by which antivenoms are assessed preclinically has received little attention.

The World Health Organisation (WHO) endorsed neutralisation of lethality assay has been the foundation of preclinical antivenom efficacy testing for over 40 years. However, the assay’s noted scientific shortcomings, ^4^^–^^6^ the ethical, and in many locations legal, obligation to implement the 3Rs (Replacement, Reduction and Refinement) in animal experimentation, and the progression of next generation envenoming therapies towards human clinical use, ^7^^–^^9^ has highlighted the need to refine this model to embrace advances in research and meet modern scientific and ethical standards. Consequently, a meeting of stakeholders in snakebite envenoming was convened to discuss the modernisation and improvement of preclinical models. This meeting was held virtually on the 26 ^th^ of January 2023 and was attended by over 100 representatives from academic institutions, international health agencies, animal welfare groups, antivenom manufacturers, clinicians, and policy makers. This article details the areas of discussion held on the day, with additional commentaries from the authors, and outlines recommended steps for improving preclinical testing of snakebite envenoming therapies in the future.

## The history of *in vivo* testing for snakebite envenoming therapeutics

An overview of the history of the development of the neutralisation of lethality murine models was presented by José María Gutiérrez (Instituto Clodomiro Picado, [ICP]). The very first recorded neutralisation of envenoming experiments were performed independently by Albert Calmette and Vital Brazil Mineiro da Campanha, among others, in the early 20 ^th^ century – this work pioneered the use of antisera as a treatment for snakebite envenoming. In a manner in which many researchers in the field today would be familiar with, both experimenters developed pre-incubation models of mixtures of venom and antivenom prior to administration via different routes in various animal species including mice, rabbits, and pigeons. ^10^^–^^20^ Both Brazil and Calmette noted that different results were obtained in different animal species, and that different pathologies were observed following different routes of venom administration. ^21^^,^^22^ By the end of the 1930’s, the use of mice became commonplace to assess the ability of antivenoms to neutralise venom induced lethality. ^23^

One of the first attempts to establish a universal standard for refinement of such testing was described by J. Ispen in 1938. ^24^ With further refinements being made by Christensen, ^25^^,^^26^ it was found that by using intravenous (i.v.) injections of antivenom and venom mixtures in mice, it was possible to standardise preclinical testing outcomes by expressing venom neutralisation as the mg of venom neutralised per ml of serum (antivenom). However, Ispen’s method was not universally adopted due to its technical complexity. A survey of existing methods to assess antivenom potency was later published by Grasset, ^27^ who highlighted the heterogeneity in implemented methodology at the time. The need for a universally consistent method for analysis of antivenom efficacy was recognised by the WHO, which held key meetings to discuss standardisation and control of antivenoms in 1970 ^28^ and 1979, ^29^ with the first globally standardised form of the assay described in 1981. ^30^ An additional meeting was held in 2001, ^31^ which paved the way for the eventual publication of the first detailed WHO Technical Report Series (TRS) regarding antivenom production and regulation in 2008 (TRS964, annex 2), with a revision in 2017 (TRS1004, annex 5), ‘ *WHO Guidelines for the production, control and regulation of snake antivenom immunoglobulins’.* ^3^^,^^32^

## The model today

The standardised murine ‘neutralisation of venom lethality assay’ (also known as the median effective dose [ED _50_] assay) has become the global standard for examining the efficacy of existing and new envenoming therapies. ^3^^,^^5^^,^^30^^,^^33^ The assay is listed as a requirement by many national regulatory agencies, and national pharmacopoeia, as proof of antivenom efficacy before a product is approved for clinical use. The assay, as described in the WHO guidelines, ^3^ is performed by mixing a fixed dose of venom with varying doses of a therapeutic agent. This dose of venom is typically three to six times the median LD _50_ (median lethal dose - the dose which induces death in 50% of mice within a group), to ensure 100% death. The mixture is then incubated for 30 minutes at 37 °C prior to i.v. or intraperitoneal (i.p.) injection into mice. Survival of the mice after a specified interval of time (24 or 48 hours, depending on route of administration) are recorded, and statistical analysis performed (Probits or Spearman-Karber) to calculate the ED _50_ –– the quantity of therapy resulting in survival of 50% of the injected mice. The median effective dose can be expressed as mg venom per mL antivenom (the most common), μL antivenom per ‘challenge dose’ of venom, or μL antivenom per mg of venom. This ED _50_ is used to predict the clinical efficacy of an antivenom against the venom for which it is indicated. More recently, the WHO has recommended that ED _50_ data be converted to potency (P), a metric which estimates the dose for complete neutralisation of lethality, rather than the ED _50_. ^34^

## The success of the neutralisation of venom lethality assay

There are several advantages to the neutralisation of lethality assay that have led to its long-standing use. Firstly, it is extremely easy to perform, not requiring any specialist equipment or expertise. Secondly, it is a ‘one model fits all’ approach, providing outputs (dead/alive) on therapeutic efficacy for all snake venoms and antivenoms despite the great diversity in venoms and their pathological effects (i.e. haemotoxicity, neurotoxicity, cytotoxicity, etc), so it can easily be applied to any new venoms being evaluated, and can generate efficacy data quickly. The success of the model has enabled substantial achievements in the field of snakebite envenoming. These include supporting the production of new antivenoms, identifying appropriate antivenoms for regions, whilst also providing evidence for serious shortcomings in antivenom provision in many regions, including sub-Saharan Africa, India, South-East Asia, and Latin America, ^35^^–^^38^ and showing the potential use of certain antivenoms against snake species for which specific antivenoms do not exist. ^39^^,^^40^

## The limitations of the neutralisation of lethality assay

Despite its success, the neutralisation of lethality assay has largely remained unchanged since its standardisation in the early 1980s. Its basic methodology, in place since the early 20 ^th^ century, has also remained unchanged, all the while scientific technological and societal expectations of animal research has progressed significantly. ^41^^–^^44^ There are three key issues with the neutralisation of lethality assay in its current form which make it problematic for widespread use in the 21 ^st^ century: ethical concerns, scientific validity, and regulatory concerns.

### Ethical issues

The ethical use of animals in scientific experimentation is not the focus of this discussion, and we point readers to interesting articles ^45^^–^^47^ and resources (e.g., www.understandinganimalresearch.org.uk, www.animalfreeresearchuk.org, www.nc3rs.co.uk) regarding this fundamental topic. Here, we are going to assume an understanding of the use of animals for development and quality control of antivenoms where non-animal alternatives are not currently available, or appropriately validated. There are two key ethical issues relating to the neutralisation of lethality assay: the number of mice used, and the level of suffering experienced, both to be taken in context of the additional ethical concern of the ultimate translatability of the obtained data (see scientific validity, below).

Mice used in the neutralisation of lethality assay often experience extreme pain, distress, fear, pathology (e.g. breathing difficulties, bleeding, organ damage, paralysis, etc) or ultimately death – like human snakebite victims. Due to the length of the protocol, suffering is often prolonged (up to 24 to 48 hours) and is often without analgesia, with the added distress of mice being left alongside dead cage mates. It should be noted that prolonged suffering without amelioration is specifically forbidden in some jurisdictions (for example in the United Kingdom and European Union) and so researchers have been required to adapt the assay to comply with relevant legislation (see r *egulatory issues*, below).

The level of suffering is further put into context when the numbers of mice undergoing this procedure are considered. The neutralisation of lethality assay consumes a significant number of animals in order to demonstrate the preclinical efficacy of an envenoming therapeutic. Typically, the assay consumes at least 5 experimental groups of 5 mice (25 mice/venom or venom/antivenom mixtures). The statistical analysis (Probits or Spearman-Kaber) requires at least one group in which all mice succumb to venom injections (an all-death group) and one group where all mice group survive, along with multiple groups with varying numbers of survivors and deaths to allow the calculation of an ED _50_ value. As antivenoms are often designed to treat envenoming caused by multiple snake species (known as polyspecific antivenoms), the assay needs repeating for each different venom for which the antivenom is indicated for. Thus, it is not uncommon to subject hundreds of mice to this procedure to assess the efficacy of a single batch of antivenom. ^33^ A study in 2020 assessing the published preclinical efficacy of antivenoms for sub-Saharan Africa estimated a figure of 3,980 mice used in the neutralisation of lethality assay across 18 publications. ^33^ It is likely that manufacturers are required to use multiples of this number each year to assure the quality and undertake batch release of their antivenom products.

### Scientific validity

Nicholas Caswell (LSTM) presented an overview of the scientific shortcomings of the current model, highlighting that the primary issue in terms of scientific validity with the neutralisation of lethality model is that, like other preclinical models, it is not reflective of the human scenario. The high failure rate (>90%) of potential therapeutics that enter clinical trials is in part due to this, alongside other factors including key differences between humans and the model species used, the use of flawed or non-clinically reflective preclinical models, which combined lead to a lack of translatability in efficacy and toxicity outcomes in clinical trials. ^48^ These have been well documented over the years, ^49^^–^^53^ and are all relevant to the current neutralisation of lethality assay, albeit have often not been as well evidenced, as snakebite envenoming therapies have thus far largely avoided these issues due to the unusual regulatory approval framework associated with their clinical use, whereby they often bypass clinical trials.

To delve deeper into the scientific validity of the model, it is important to discuss the nature of the clinical envenoming scenario, and how this differs from the existing pre-clinical scenario. Human snakebite envenoming typically involves intra-dermal (i.d.), subcutaneous (s.c.) or intra-muscular (i.m.) venom injection, with antivenom administered i.v. hours later in a hospital. The current model is a an artificial ‘best case scenario’, whereby an artificially large and above lethal quantity of venom and therapy are premixed and incubated before injection directly i.v. or i.p. There are several key points that make this problematic in terms of predicting the therapeutic efficacy of snakebite treatments:

1) The current murine administration routes, which entails of a single bolus injection, results in an instantaneous (i.v.) or rapid (i.p.) concentration of toxins in the bloodstream, as opposed to a steadier, sustained release of toxins from the bite site in patients. ^54^^–^^56^ As such, whilst providing a readout of the capacity of an antivenom’s ability to neutralise a fixed dose of venom, the assay does not consider the pharmacokinetic or pharmacodynamic properties of either the venom or the therapy being tested. ^57^^–^^59^
2) The high dose of venom administered (above lethal) typically rapidly overwhelm the model (i.e., cause lethality in minutes) and so the model is unable to mimic delays in therapy administration which always occur in human treatment.
3) Whilst many snake venoms can cause lethality, some medically important venoms do not. An obvious and commonly cited example is that of cytotoxic African spitting cobra venoms. ^5^^,^^33^ These rarely cause fatalities in humans, although they do cause substantial local effects, i.e., dermonecrosis. ^60^^,^^61^ It is likely that neutralization of lethality observed in experiments using spitting cobra venom in mice is inferring the neutralisation of a venom component which has very limited clinical translation. This poses the ethical question of when this assay should be employed, and whether it is appropriate to use a lethality assay for certain venoms which rarely cause human fatalities. It further emphasises the need to adapt animal models that are better representative the clinical manifestations of snakebite envenoming, which may in turn identify more appropriate treatments.

Ultimately, due to the fundamental issues above, the neutralisation of lethality assay is not appropriate to use to perform extrapolations to human envenoming contexts. However, influential documents and policies, such as the WHO Target Product Profiles (TPP) and antivenom risk benefit assessments, have recommended such extrapolation to inform antivenom choice and dose (albeit in the absence of a more appropriate model in which to do this). ^34^ This approach ultimately does not take into consideration the reality of complex pharmacokinetic and pharmacodynamic interactions of individual venom components and therapies in complex multi-tissue and organ environments – henceforth, the current model is too simplistic to make such important, accurate dose predictions. Moreover, differences in the susceptibility to venoms of rodents and humans also question the validity of such extrapolations. ^62^^,^^63^ It is notable that the complexity of envenoming is used as a justification of mouse use, in addition to testing the ‘worst case scenario’ (death), but this is overlooked when considering dose regimes.

Clearly an antivenom which fails to neutralise venom in the best-case scenario of venom and therapy co-incubation and administration in the neutralisation of lethality assay is not going to be suitable for treating envenoming clinically. However, it is imperative to stress that if an antivenom appears effective in this assay, it does not mean that this efficacy will translate to the clinical scenario, likely due in part to the scientific limitations limited listed above. The issues with the assay’s scientific validity in the context of the severe welfare costs, makes the ethical justification of the model increasingly challenging by today’s standards.

### Regulatory issues

Regulatory issues consider both experimental animal use regulations and pharmaceutical approval legislation. There is a push from many national funding agencies towards greater scientific scrutiny and justification of animal usage, which is in direct conflict with antivenom manufacturers, many of which are bound by pharmacopoeia or national regulatory agency guidelines that specify the use of the existing murine model and the current WHO guidelines. This is further compounded by the development of new generations of envenoming therapeutics which will likely be required to enter through the traditional clinical regulation pathways, and thus will require much more rigorous preclinical *in vivo* testing and data generation. ^64^

***Animal experimentation regulation***

Animal experiment regulation standards vary substantially across the globe, with different levels of stringency depending on legislations in particular jurisdictions. ^65^ The neutralisation of lethality assay in its basic form, as outlined in the current WHO guidelines, ^3^ can no longer be used in several regions without substantial modification or adaptation. Under the UK Animals in Scientific Procedures Act 1986, and the EU Directive 10/63/EU, ^66^ it is a legal requirement that death as an experimental endpoint and long-lasting pain and suffering which cannot be ameliorated, must be avoided as far as possible. In scenarios where death is considered unavoidable, such as the neutralisation of lethality assay, as inferred by its name, experiments must be designed to limit death resulting from the experiments, and should strive to end suffering as soon as feasible through the implementation of humane endpoints and euthanasia. These requirements have resulted in researchers being legally required to implement various modifications and adaptions in order to comply with local regulations (see “improvements to the standard model”, below). This in turn is contributing to an ever-increasing divergence from the global harmonisation of the assay which the WHO guidelines attempts to achieve. ^33^ As improvements in animal research techniques and welfare continue at pace across the medical health research field, the continued justification of using the neutralisation of lethality model to both regulatory authorities, and increasingly funders, will continue to become ever more challenging and its use ever more restrictive.

***Pharmaceutical regulation***

A major reason for the lack of revision of the neutralisation of lethality assay are requirements by geographical or national regulatory agencies specifying the use of the assay for a product to be registered. Changing assays is a laborious and costly process which requires detailed validation, and thus is not performed without a compelling justification. Ultimately, antivenoms are overwhelmingly produced by small-medium companies or public entities with restrictive budgets which generally do not have the resource or expertise to investigate alternatives. Academics developing new therapies and scrutinising existing therapies ultimately want to use similar models to the manufacturers to assist with translation of findings, so are disincentivised to using alternative models.

However, regulators and manufacturers are committed (and bound by relevant national legislation) to implementing the 3Rs, which many examples of improved methodology have been adopted by pharmacopoeias in the recent past, most notably for biologics. ^67^^,^^68^ This is reflected by some antivenom manufacturers actively investigating alternative efficacy models, including *in vitro* and non-regulated *in vivo* modes, for product release due to the ethical challenges and costs associated with conventional ED _50_ testing. Examples include the ongoing development of an embryonated egg model for testing by Seqirus, ^69^^–^^71^ and approval in recent years of antivenomics (immunoaffinity chromatography) techniques for product release by a national regulator, ^72^ and as screening test for antivenom efficacy before proceeding into mouse assays. ^3^

***Regulation of next generation envenoming therapies***

Diogo Martins and Alethea Cope (Wellcome) provided an insight from a regulatory perspective, and highlighted how antivenoms are an anomaly in the therapeutic development pipeline. The standard pathway for drug development is complex, expensive, and lengthy (generally 10-15 years in duration), and depends on lead discovery, preclinical testing, clinical trial phases followed by National Drug Agency review and approval. This process requires the provision of appropriate information packages and dossiers which ensure a high-quality product. Historically (and even currently), many antivenoms have not been required to undergo Phase I-III clinical trials, meaning that the typical therapeutic development pipeline is truncated, with national drug association/regulation approval often occurring after initial preclinical testing. As >90% of novel drugs progressing through the standard therapeutic development pipeline fail in human clinical trials, blamed in part to unsuitable preclinical models, it is perhaps unsurprising that there have been occurrences of suboptimal antivenoms reaching patients and providing to be ineffective. ^73^^,^^74^ Until very recently, there has been an expanding consensus that antivenoms should undergo clinical trials, ^75^ however, due to the cost of such trials and regulatory shortfalls, this consensus has started to wane, with some stakeholders now in favour of Monitored Emergency Use of Unregistered and Investigational Interventions (MEURI) assessments, ^76^ instead.

However, next generation envenoming therapies, such as small molecule inhibitors and monoclonal or recombinant antivenoms, which are under development as possible alternatives or adjuncts to conventional antivenom therapy in recent years, are highly unlikely to be able to enter the same regulatory pipeline as antivenom. These new therapeutic candidates will likely be subjected to appropriate full therapeutic development and regulatory pipelines, being required to proceed through preclinical and clinical assessment before approval. ^62^^,^^64^

## Improvements to the standard model: Refinement and Reduction

With the above issues in mind, several refinements to the standard neutralisation of lethality assay have been developed in recent times.

Gabriela Solano (ICP) described two modifications to the model which have recently been implemented at the antivenom manufacturer ICP. The first refinement was the use of analgesia for all *in vivo* envenoming assays, including the neutralisation of lethality assay, which is now common practice not only at ICP but at LSTM, too. The WHO guidelines “strongly recommends” the use of analgesia to reduce pain and distress in mice, however, it is not described in the methodology, and anecdotally seems to not be implemented widely. The analgesic of choice at ICP is tramadol, while morphine is used routinely at LSTM, with the choice of drug being dependent on availability and accessibility in specific countries, and all mice pre-dosed subcutaneously (s.c.) 15 minutes prior to venom administration. Importantly, the use of analgesia has been validated in the context of experimental outcome, with the results demonstrating analgesia use proved effective at reducing pain scores in mice, without adversely affecting lethality or neutralisation data. ^21^^,^^77^^,^^78^

The second refinement was focused on the study design of the neutralisation assays. The current WHO guidelines for the neutralisation of venom lethality assay outlines a 24-hour timeframe for pre-mixed i.v. injections, or a 48-hour timeframe for pre-mixed i.p. injections. ICP investigated whether this timeframe could be shortened, reducing the length of time in which mice are suffering the effects of venom. The reduction in time was evaluated by calculating the LD _50_ of venoms and ED _50_ of antivenoms at 6-, 24-, and 48-hours post injection using a wide variety of venoms and two poly-specific antivenoms. The results demonstrated a significant correlation between LD _50_ and ED _50_ values estimated at the three observation times, with the majority of deaths occurring within the initial 6-hour timeframe. ^79^ Based on similar observations, LSTM have also been using a reduced 6-7 hour model for all envenoming and neutralisation studies for over a decade. ^36^^,^^80^

To our knowledge, very limited attempts have been made to reduce the number of animals used, with justification inferred due to the statistical requirement of specified numbers for analysis. However, a modified version of the neutralisation assay which substantially reduces the number of mice required to infer efficacy of an antivenom has been attempted in recent years and was presented by George Omandi (K-SRIC). In a 2017 study, a ‘gold standard’ approach to the neutralisation of lethality assay was used, which involved examining the ability of test antivenoms to prevent lethality at half, equal or 2.5 x the volume of the current antivenoms which provide 100 % venom neutralisation and survival (2 x ED _50_ dose volume). ^37^ This approach, whilst still requiring the conventional neutralisation of lethality assay to determine the ED _50_ of the two arbitrarily chosen gold standard antivenoms, enabled an analysis of the efficacy of four regionally marketed antivenoms vs. six regional venoms using 75 % fewer the number of mice which would be required if using the standard assay (150 vs. 600). ^37^

## Rescue models

The use of post-envenoming (‘rescue’ or ‘challenge then treat’) models have increased in frequency in recent years as the limitations of the standard neutralization of lethality model have become more apparent. These models involve an initial injection of venom followed by an independent, and often time delayed, administration of therapeutic – a scenario much more reflective of human snakebite envenoming. The assay allows venom and therapeutics to be administered via different routes, allowing for a range of pharmacokinetic properties to be mapped and a more accurate output of potential therapeutic efficacy. As mentioned above, these models are especially useful for assessing the preclinical efficacy of next generation therapeutics, and thus represent a refinement in terms of scientific validity. ^6^ However, the current drawback to rescue models is that they often still rely on lethality (unless specifically testing using a localised model) and still require death as an endpoint for determining effectiveness, and may possibly induce a greater degree of suffering (due to administration of venom with therapy withheld for a period).

Furthermore, large differences in drug neutralizing potential can be observed in pre-incubation and rescue models, as demonstrated by Christoffer Sørenson (DTU). He explained that a candidate anti-myotoxin mAb was effective in reducing myotoxicity when pre-incubated with a myotoxin or *B. asper* venom, but potentiated myotoxicity, when administered in a rescue model of envenoming. i.e., after venom injection. ^81^ This demonstrates clearly how the current standard neutralization of lethality assay can overestimate therapeutic potential, which may possibly lead to failure during clinical trials, and the potential utility and importance of rescue models, which attempt to better recapitulate a clinical envenoming, albeit with limitations. Perhaps of most concern, is that conventional antivenoms have scarcely been used in such rescue models, thus no benchmark yet exists what efficacy looks like using conventional treatment. and such models do not yet follow a universal standardised approach. In summary, rescue models must be performed with analgesia and careful monitoring, alongside the development and application of humane endpoints to avoid death and limit suffering.

## What future *in vivo* models of envenoming could look like: ending the reliance on lethality testing

An example of a condition which has a similar capacity to make a mouse as comparatively rapidly critically unwell as snakebite envenoming is sepsis. Lethality was once a common readout in sepsis models but there is a general consensus within the field that this is unjustifiable, due to the binary live/dead readouts not being useful for determining the clinical potential of a therapy, and the harm:benefit assessment of such studies. ^42^ Manasi Nandi (Kings College London) described how technological advances in imaging and cardiac monitoring, and the implementation of biomarkers, have been established which allow comprehensive analysis of the efficacy of interventions in sepsis before mice become critically ill and long before they reach lethality in such models, whilst highlighting the wealth of data that can be obtained as compared to a simple death/survival result. ^42^^,^^82^^–^^85^ This example demonstrates that the identification of appropriate surrogate markers can allow for the implementation of humane endpoints in animal models, which would otherwise progress to death as an endpoint, whilst still meeting the scientific aims and objectives of the study.

As there are several parallels with the previous sepsis mouse models, a fundamental rethink, and harm:benefit assessment of the current neutralisation of lethality assay is required. Is it essential that we need to assess the ability of an antivenom to prevent lethality specifically, when in fact the key question is to evaluate an antivenom’s ability to neutralise or reverse envenoming pathology which *may* lead to lethality if left to run its course, and thus, is death really required as an endpoint? Could a refined model be explored, and biomarkers be implemented at earlier stages negating the need to run studies until death, as has been done with the refined sepsis models? For example, if in the neutralisation of lethality assay, in which therapy and venom are premixed, a neurotoxic venom causes limb paralysis in the presence of antivenom, is that not an informative readout of the inefficacy of a therapy? Is it ethical to then leave an animal under study to further deteriorate and suffer from limb paralysis to severe respiratory distress, to eventual death in order to demonstrate therapy failure? A similar argument can be made in the context of haemotoxic envenomings and whether early murine readouts such as coagulopathy must progress to systemic internal (and lethal) haemorrhage to be viewed as ‘useful’ readouts. The latter is particularly relevant when considering that of the few robust clinical trials and studies of antivenom efficacy performed to date; several have used measures of coagulopathy as study endpoints, ^86^^–^^88^ and would incorporating such markers into preclinical studies improve the translatability to clinical testing? Equally, if an experimental animal survives the duration of the study but is critically unwell at experiment end, is it appropriate to consider a therapy effective, based on a simplistic binary outcome of alive or dead? Similar questions have been asked in other fields, such as do vaccines need to prevent lethality or simply illness? ^89^ Such questions call for novel, data-rich, informative approaches to assess venom toxicity and therapeutic efficacy, and a review of current practices.

Due to legal requirements under UK legislation, humane endpoints have been established by researchers at LSTM and implemented for over a decade during neutralisation of lethality studies. ^90^ Humane endpoints are put in place to prevent and minimise pain and distress, and as a substitute for death. Death as an endpoint is now rarely accepted, and researchers must present conclusive evidence to support the use of such an endpoint, and are essential in studies where potential severe suffering, or death, may occur, such as acute toxicity studies, infectious disease models, or models of cancer or neurodegenerative diseases. ^91^ The endpoints used at LSTM during neutralisation of lethality studies are implemented when mice are severely moribund (e.g. loss of righting reflex, seizure, signs of external haemorrhage, dyspnoea) and enable a reduction of the duration for which prolonged suffering occurs during experimentation. Although a refinement to the model in the context of expeditiously ending animal suffering, the use of such endpoints in this model has proved more contentious, and have not been implemented in other settings. The primary reasoning for this is the concern over the subjective nature of implementing endpoints, and the potential for inaccurate reporting of an antivenom’s efficacy, and the consequent potential negative impact on human envenoming outcomes due to lack of clinical trials. Although termed ‘humane endpoints’, there is also the argument to be made that leaving an animal until a moribund state cannot be regarded as ‘humane’, as animals will still have suffered severe pain and distress to reach this state. ^92^ Thus, a global standardisation of humane endpoints would improve animal welfare substantially.

It is clear from multiple clinical studies that different venoms cause different pathologies. Lethality may ultimately be a common pathology of envenoming caused by many snake species, but it is a result of the different toxic effects induced by individual venoms, which in turn consist of multiple toxins. Like cancer, snakebite should not be viewed as a single disease, but as a series of diseases with distinct pathologies under an umbrella term. Indeed, a recently published clinical outcome set for future snakebite therapy clinical trials, ^88^ and a document outlining TPPs for conventional antivenoms for use in sub-Saharan Africa, ^34^ detail pathology specific measurements that should be used as outcomes in clinical trials (such as international normalised ratio (INR) for coagulopathy) and clinical thresholds antivenoms should meet (such as persistence of coagulopathy after a defined time frame). Currently, pathology specific preclinical assays, when available, are described as ‘supplementary’ and typically overlooked, but would be useful in providing readouts of efficacy as opposed to only a live-dead phenotype, and provide a more informative data set prior to progression into clinical trials (discussed below).

To develop more appropriate pathology specific models, it would therefore be sensible to encourage a closer cross-talk between colleagues working on both sides of the preclinical/clinical divide, to facilitate development of preclinical testing methodologies to better support the development of initiatives like TPPs and clinical trial outcome sets. Below we briefly describe examples of what better developed models that avoid lethality, but could better support future antivenom development and assessment, could look like.

*Echis* spp. venoms: It is clear from clinical studies that the main effects induced by *Echis* venoms are haemorrhage and coagulopathy. ^86^^,^^93^ Therefore, instead of using the neutralisation of lethality assay, an alternative approach would be to assess neutralisation of venom induced coagulopathy and haemorrhage. The WHO listed minimum venom defibrinogenating (coagulopathy marker) and haemorrhagic dose assays are one hour and 2-3 hours, respectively, in duration and involve using comparatively lower doses of venom compared to the neutralisation of venom lethality assay, thus resulting in lesser suffering in mice. ^3^ Although these tests are typically conducted with preincubation of venom and antivenom, they can be easily adapted to a rescue model format where needed, with appropriate validation.

*Bothrops* spp. venoms *:* Clinical observations have shown that relevant effects of many *Bothrops* envenomings are haemorrhage, coagulopathy, and myonecrosis. ^94^ Thus, the assessment of haemorrhage and coagulopathy, as described for *Echis*, could potentially be applied, while myonecrosis can also be assessed *in vivo*, by measuring the increase in plasma creatine kinase activity as a surrogate marker for myotoxicity. ^95^ Although this test involves pain, the duration of study is reduced to 3 hours, and analgesia can be used to reduce pain.

Cytotoxic African *Naja* spp. venoms: Clinically these venoms induce cutaneous necrosis (dermonecrosis), while lethality is less frequent. ^60^ As has been repeatedly stated over the years, the use of the neutralisation of lethality assay is arguably inappropriate in this case; however, it persists due to the current guidelines and regulations. Instead, neutralization of local dermonecrosis would be more relevant. Similarly, to the minimum venom defibrinogenating dose assay, pathology specific assays for necrosis do exist (the neutralization of venom necrotizing activity) but are problematic in that they are extremely painful and prolonged. To develop clinically relevant neutralisation of venom dermonecrosis activity, much could be learnt from preclinical models in related fields, such as those studying necrotizing soft tissue infections, burns and hypoxia. Likewise, dermonecrosis may be modelled with cytotoxicity assays in cell culture models or organotypic models of human skin. ^60^ However, care must be taken in this approach, as recent research has suggested that cell culture models do not accurately recapitulate dermonecrosis. ^96^

*Daboia* spp. venoms: The pathophysiology of envenomings by *Daboia* spp. is highly complex as it involves, in addition to lethality, a variety of effects such as haemorrhage, shock, coagulopathy, acute kidney injury and systemic capillary leakage syndrome. ^97^ In addition to the tests for defibrinogenation and haemorrhage mentioned above, the systemic capillary leakage syndrome can be followed by determining haematocrit, ^98^ and kidney injury can be assessed by quantification of serum creatinine and urea levels. ^99^ In both cases, these tests can be done at early time intervals thus reducing the duration of animal experimentation.

Our intricate knowledge of venom composition and pathophysiology has increased substantially in the past two decades. *In vitro* biochemical and cell-based assays, and more recently *in silico* assessments, ^58^^,^^100^ are now routinely being developed and used based on our understanding of the main mechanisms of venom action, with the objective of rapidly assessing the neutralising capabilities of potential therapeutics in the context of clinically relevant bioactivities, and to improve the chances of progression to clinical use. ^101^^–^^105^ The ideal setting would be the development of *in vitro* assays with direct correlation to outputs gained from *in vivo* lethality and venom neutralisation assays, which could potentially provide a replacement to *in vivo* models, or at the very least, a reduction in the number of animals subjected to the neutralisation of lethality assay. However, such approaches must be validated on a case-by-case basis since each venom-antivenom system behaves differently. In some cases, immunochemical assays have shown correlation with lethality. ^106^ In other cases, neutralisation of *in vitro* venom activities (such as coagulant activity on plasma) correlate with lethality. ^107^ Lethality induced by neurotoxic venoms with a post-synaptic mechanism of action can be modelled with an assay based on the inhibition of the venom binding to or activation of cholinergic receptors. ^101^^,^^102^ In other cases, predictions of venom therapeutic efficacy made from *in vitro* assaying have not translated into *in vivo* preclinical venom neutralisation. ^108^ Once validated appropriately, examples of such could be used as a first point testing call, made clear within policies to be prior to *in vivo* assays, and would facilitate in efforts to minimise animal use, or at the very least only allow for therapeutics with a high confidence of predicted efficacy *in vivo*, entering such studies.

## Risks of moving away from the neutralisation of lethality assay

It is easy to propose that models need to be better, more refined, and provide more data, in order to reduce the animal welfare cost and to increase the translatability of therapeutic efficacy into the clinic. However, the current realities of antivenom manufacturing must be taken into account. David Williams (WHO) emphasised that snakebite is a neglected tropical disease, that antivenom is an incredibly expensive drug to manufacture, and the economic incentives for private pharmaceutical companies are poor, whilst national public manufacturers, especially in resource limited settings, have limited budget or access to the highly skilled workforce required for performing such *in vivo* experiments, or indeed complex non-animal alternatives. Thus, any changes to the preclinical regulation of antivenoms must be proportionate, affordable, and sustainable and not inadvertently result in antivenom manufacturers withdrawing or being left unable to contribute to this already vulnerable and fragmented market.

## The changing landscape of animal research and testing of biological therapeutics

This workshop was held against a background of considerable debate and change in the scientific community around the translational value of animal models. ^109^^,^^110^ In parallel, there has been increasing research into novel non-animal methodologies/technologies (often referred to as NAMs and NATs) that have the potential to replace *in vivo* assays in a variety of area. ^49^ There is widespread acknowledgement that transition towards non-animal approaches is not only ethically desirable but scientifically superior in many cases with generally lower variability and higher sensitivity. *In vitro* approaches are frequently more mechanistically relevant and quantitative than phenotypic, ‘black box’, mechanistically agonistic animal models. In the field of biologics, vaccines in particular, there has been considerable effort to transition away from animal models for quality control batch release testing. The European Pharmacopoeia took a considerable step towards encouraging the adoption of *in vitro* test methods with the publication of chapter 5.2.14 in January 2018. ^111^ This chapter not only provides guidance to facilitate implementation of *in vitro* methods as substitutes for existing *in vivo* methods, but specifically indicates that because critical quality attributes for a given vaccine are likely to be assessed differently by *in vivo* and *in vitro* assays, it may not be scientifically justified to demonstrate agreement between these two approaches as part of the validation of an *in vitro* alternative assay. Given the current regulatory appetite for, and momentum towards, adoption of non-animal approaches in other biological therapeutics, coupled with the clear ethical issues with the current lethality assays, it is a good time to renew efforts to develop non-animal test methodologies for antivenom potency testing.

## Conclusions and short-term suggestions

It was widely agreed amongst the contributors to the workshop that the existing snakebite preclinical development and testing approach needs substantial modernisation and improvement, and that in its current form is not suitable to support the development of the new generation of envenoming therapies. With drug development programmes for snakebite currently underway, and clinical trials for antivenom and alternative therapeutics on the horizon, it is evident that an updated *in vivo* models, and better *in vitro* and *ex vivo* alternatives, are urgently required to provide more information to support such developments and facilitate therapeutic candidate selection, in addition to improving the current harm:benefit assessment, and animal suffering encountered in the current model. An increased crosstalk between clinicians, experimental scientists, antivenom manufacturers and regulators is likely to benefit this field, catalysing the development of alternative ways to assess antivenom preclinical efficacy, that is more realistic and predictive of the clinical scenario. Moreover, as regulatory pressures increase, or as more countries modernise animal welfare regulation, the countries in which the lethality assays can be performed will decrease, thus underscoring the need for an acceleration of replacement, animal-free, alternative testing research for antivenoms. In the meantime, whilst possibly difficult for manufacturers to implement changes due to regulatory requirements for product release, there seems little justification to argue against the implementation of multiple small refinements to the neutralisation of lethality assay (analgesia at least, reduction in time and humane endpoints if possible), which will substantially increase animal welfare. ^112^ We recommend these refinements are incorporated explicitly, within the sections describing the methodological details of this assay, in future revisions of the WHO guidelines.

Despite the substantial animal costs outlined in this article, in the absence of the requirement for antivenoms to undergo the typical pathway for demonstrating clinical effectiveness through human clinical trials, the harm:benefit assessment these assays remains greatly skewed in the favour of human health benefit, rather than animal welfare. However, this does not mean improvements cannot be made. Ultimately, all snakebite envenoming stakeholders, from victims to manufacturers to researchers, owe a great deal to the consumption and fate of the tens of thousands of mice consumed in the neutralisation of venom lethality assay each year. The absolute minimum improvements that can be made as a community, for as long murine testing is necessary to demonstrate efficacy of envenoming therapies, is to try and reduce the severity of these studies and improve mouse welfare. It is important to note that refinements will not only improve the welfare of animals used in these experiments, but they are likely to also improve the quality and validity of the science being performed, and therefore improve outcomes for human snakebite victims, too.

## Data Availability

No data are associated with this article.
