# Supplementary material for: Improving  in vivo assays in snake venom and antivenom research: A community discussion
Source: F1000Res. 2024 Mar 15;13:192. [Version 1] doi: 10.12688/f1000research.148223.1 (PMC11066530; doi:10.12688/f1000research.148223.1)
Supplement: Peer review history

# Reviewer report: Reviewer response for version 1

**DOI:** 10.5256/f1000research.162509.r265200

**Published:** 2024-05-14

**Authors:** Karen de Morais-Zani

**Affiliations:** 1 Instituto Butantan, São Paulo, Brazil

This opinion article describes the topics presented and discussed at the meeting “Improving in vivo snake venom research” (2023), organized by the Center for Snakebite Research and Interventions of Liverpool School of Tropical Medicine (UK). 

 In the manuscript, the use of the murine model to determine the lethality of snake venoms and its neutralization by antivenoms was discussed, as well as suggestions for refining the model, with the aim of meeting the 3Rs principle in the use of animals. The authors also gather references that support the discussion of the topic and the proposed adaptations/modifications. This is a topic of great relevance not only for those directly involved in the area, but for the scientific community in general.

 Major points:

 The authors describe the standardized murine model for neutralization of venom lethality and comment on the advantages of the assay that led to its success/widespread use. However, the manuscript focuses on discussing the limitations of the model, especially regarding its ethical issues and scientific validity. Refinements to the standard neutralization of lethality assay were described, as well as a modified version with reduced number of mice required. Importantly, the need and the challenges of the implementation of humane endpoints are discussed.

 Minor points:

 The authors give an historical overview of the development and standardization of the murine model for accessing the snake venom lethality and its neutralization.

 Additionally, authors give examples of alternatives to lethality model considering the pathophysiology of the envenomation caused by genera *Echis*, *Bothrops*, *Naja* and *Daboia*.

 I only suggest a review by the authors to amend some writing/typing mistakes.

Is the topic of the opinion article discussed accurately in the context of the current literature?

Yes

Are arguments sufficiently supported by evidence from the published literature?

Yes

Are all factual statements correct and adequately supported by citations?

Yes

Are the conclusions drawn balanced and justified on the basis of the presented arguments?

Yes

Reviewer Expertise:

Compositional and functional characterization of snake venoms.

I confirm that I have read this submission and believe that I have an appropriate level of expertise to confirm that it is of an acceptable scientific standard.

# Reviewer report: Reviewer response for version 1

**DOI:** 10.5256/f1000research.162509.r259966

**Published:** 2024-05-02

**Authors:** Jarrod Bailey

**Affiliations:** 1 Physicians Committee for Responsible Medicine, Washington, DC, USA

The authors are to be congratulated on putting together such a comprehensive, balanced, critical, constructive and well-referenced review of an ethically and scientifically difficult topic.

 They deal admirably and honestly with an appraisal of the history and current use of animals in envenoming research, development and testing, including detailed issues with ethics and scientific validity, particularly with regard to the neutralisation of lethality assay.

 Of particular interest to the scientific community may be the lack of human relevance and translatability of snakebite envenoming research and related therapeutic development which, when combined with the severity of many of the procedures in which animals are used as experimental/testing subjects, raises a formidable case that the justification of animal use in these ways must be untenable.

 The open and honest nature of the authors’ appraisal is of the type that can take some courage, particularly when it comes to critical views of established animal models. They also present a commendable and welcome account of ‘Three Rs’ approaches, which reflects a wider appreciation of the welfare issues they detail. The need for action, in terms of more focus on reduction, and most certainly on replacement, is also discussed.

 Of particular note in this regard is the section of the manuscript “ *What future in vivo models of envenoming could look like: ending the reliance on lethality testing*.” Here, and in their Conclusions and Suggestions the authors deal well with the issues of *Refining* lethality testing, but do not shy away from discussing the need for more focus on *Replacing* *in vivo* assays, both for welfare/ethical and scientific/translational reasons. They cite examples of progress that have been made in this respect, and acknowledge the need to accelerate this progress in order to better address the animal welfare issues involved in *in vivo* methods. They also discuss the anticipation of regulatory pressures to replace animal testing in this field, based on increasingly stringent animal welfare regulations, which may increase the urgency for replacement.

 The authors could add a couple of notes to improve these discussions which I believe are pertinent, which would be relevant to their discussions of *Refinement* versus *Replacement*, and their assertions that any *in vitro* replacement methods should be validated on case-by base bases. In a paper I have currently *in press* (Ref [1]), I argue that focusing on *Refinement*, whatever the merits, can distract from and even discourage efforts on *Replacement*, which is significantly more urgent and preferable, both ethically and scientifically. Further, they might wish to briefly discuss the validation process. This process can take years, and is frustratingly and unnecessarily time consuming when there is an urgent need for a new, more translational and human-relevant method. Some argue that it is therefore important to ensure that the validation ‘bar’ is not set too high, and that an ethically superior method does not have to be perfect – it just has to match, or slightly improve on the performance of an animal based method to be acceptable.

 I highly recommend the approval of this manuscript without delay, so it can elicit important discussions within the field which should promise more ethical, human-relevant, predictive and rigorous science, as well as more generally in other biomedical areas.

Is the topic of the opinion article discussed accurately in the context of the current literature?

Yes

Are arguments sufficiently supported by evidence from the published literature?

Yes

Are all factual statements correct and adequately supported by citations?

Yes

Are the conclusions drawn balanced and justified on the basis of the presented arguments?

Yes

Reviewer Expertise:

Animal models, in vitro research methods, NAMs, molecular biology.

I confirm that I have read this submission and believe that I have an appropriate level of expertise to confirm that it is of an acceptable scientific standard.

## References

[1] : It’s Time to Review the Three Rs, to Make them More Fit for Purpose in the 21st Century. *Alternatives to Laboratory Animals* .2024;52(3) : 10.1177/02611929241241187 155-165 10.1177/02611929241241187 39363487

# Reviewer report: Reviewer response for version 1

**DOI:** 10.5256/f1000research.162509.r259964

**Published:** 2024-04-29

**Authors:** Sébastien Larréché, Jean-Philippe Chippaux

**Affiliations:** 1 Département de biologie médicale, Hopital d'Instruction des Armees Begin (Ringgold ID: 56524), Saint-Mandé, Île-de-France, France; 2 UMR-S1144, Universite Paris Cite (Ringgold ID: 555089), Paris, Île-de-France, France; 3 IRD MERIT, Universite Paris Cite (Ringgold ID: 555089), Paris, Île-de-France, France

This opinion article summarizes the conclusions of an international webinar on modernizing and improving animal models in venom and antivenom research. This discussion seems indeed necessary in the context of the 3Rs aimed to improve animal welfare.

 A first chapter presented the history of lethality neutralization tests in mice, particularly during the discovery of passive immunotherapy which opened the way to the manufacture of antivenom.

 We recommend here that the authors recall that Phisalix and Bertrand were the first to report the transfer of immunity against viper venom from a previously immunized animal to another naive one, shortly preceding Calmette during the same session of the Society of Biology on February 10, 1894 (Ref-9). It is also worth noting that Calmette was the first to use antivenom in humans to treat snakebite envenomation.

 The median effective dose assay in mice as currently recommended by the WHO, and its advantages were then presented. The limitations were mentioned: ethical issues (important number of mice used, and level of suffering experienced), scientific validity and regularity issues. However, another limitation comes from the use of parametric statistical tests, which require a larger number of animals than non-parametric tests, but are unfortunately not yet accepted by drug regulatory authorities. Their use would make it possible to drastically reduce the number of animals used in neutralization tests. We noticed an error in the name of one of the contributor: “Caswell” instead of “Casewell” (at the bottom of page 4).

 Possible improvements regarding refinement and reduction, the rescue model and future developments were then presented. We fully agree with the authors’ suggestions. In addition, the potential interest of in vivo assays using large animals ( *i.e.,* sheep) deserves to be reported here as it allows to avoid certain limitations in the pharmacokinetics and pharmacodynamics of venoms and antivenoms mentioned in the webinar (Neri-Castro E,et.al., 2020 [Ref 1]) ; Paniagua D, et al.,2019 [Ref 2]).

 For the assessment of coagulopathy, it should be noted that the prothrombin time or INR must be measured according to a standard specific to the animal used. Alternatively, the use of viscoelastic tests such as TEG or ROTEM allows a global assessment of hemostasis, *i.e., platelets*, coagulation factors and fibrinolysis with a single test (Nielsen VG,et.al.,2023 [Ref 3]) ; Camacho E, et.al.,2023 [Ref 4]) ; Larréché S, et.al.,2023 [Ref 5]).

 Likewise, the study of the neutralization of venom procoagulant effect by an antivenom can be designed using an *in vitro* model, as has been demonstrated with *Echis* or *Bothrops* venoms (Rogalski A, et. al., 2017 [Ref 6]); Bourke LA, et. al.,2021 [Ref 7]).

 Finally, the authors should emphasize the urgent need for human clinical trials to validate the efficacy and safety of antivenoms, provided that standardized evaluation criteria are used (Abouyannis, et.al., 2023 [Ref 8]) .

Is the topic of the opinion article discussed accurately in the context of the current literature?

Yes

Are arguments sufficiently supported by evidence from the published literature?

Yes

Are all factual statements correct and adequately supported by citations?

Yes

Are the conclusions drawn balanced and justified on the basis of the presented arguments?

Yes

Reviewer Expertise:

toxinology, microbiology

We confirm that we have read this submission and believe that we have an appropriate level of expertise to confirm that it is of an acceptable scientific standard.

## References

[1] : Neotropical Rattlesnake (Crotalus simus) Venom Pharmacokinetics in Lymph and Blood Using an Ovine Model. *Toxins (Basel)* .2020;12(7) : 10.3390/toxins12070455 10.3390/toxins12070455 32708875 PMC7405010

[2] : Antivenom effect on lymphatic absorption and pharmacokinetics of coral snake venom using a large animal model. *Clin Toxicol (Phila)* .2019;57(8) : 10.1080/15563650.2018.1550199 727-734 10.1080/15563650.2018.1550199 30773936

[3] : Novel Toxicodynamic Model of Subcutaneous Envenomation to Characterize Snake Venom Coagulopathies and Assess the Efficacy of Site-Directed Inorganic Antivenoms. *Int J Mol Sci* .2023;24(18) : 10.3390/ijms241813939 10.3390/ijms241813939 37762243 PMC10530349

[4] : Neutralization, by a polyspecific antivenom, of the coagulopathy induced by the venom of Bothrops asper: Assessment by standard coagulation tests and rotational thromboelastometry in a murine model. *Toxicon* .2023;234: 10.1016/j.toxicon.2023.107301 107301 10.1016/j.toxicon.2023.107301 37741576

[5] : Bothrops venom-induced hemostasis disorders in the rat: Between Scylla and Charybdis. *PLoS Negl Trop Dis* .2023;17(11) : 10.1371/journal.pntd.0011786 e0011786 10.1371/journal.pntd.0011786 38011218 PMC10703418

[6] : Differential procoagulant effects of saw-scaled viper (Serpentes: Viperidae: Echis) snake venoms on human plasma and the narrow taxonomic ranges of antivenom efficacies. *Toxicol Lett* .2017;280: 10.1016/j.toxlet.2017.08.020 159-170 10.1016/j.toxlet.2017.08.020 28847519

[7] : Pan-American Lancehead Pit-Vipers: Coagulotoxic Venom Effects and Antivenom Neutralisation of Bothrops asper and B. atrox Geographical Variants. *Toxins (Basel)* .2021;13(2) : 10.3390/toxins13020078 10.3390/toxins13020078 33499001 PMC7911261

[8] : A global core outcome measurement set for snakebite clinical trials. *Lancet Glob Health* .2023;11(2) : 10.1016/S2214-109X(22)00479-X e296-e300 10.1016/S2214-109X(22)00479-X 36669810 PMC7617674

[9] : Paths to the discovery of antivenom serotherapy in France. *J Venom Anim Toxins Incl Trop Dis* .2016;22: 10.1186/s40409-016-0074-7 20 10.1186/s40409-016-0074-7 27279829 PMC4898362
